# Chemical communication at the synthetic cell/living cell interface

**DOI:** 10.1038/s42004-021-00597-w

**Published:** 2021-11-25

**Authors:** Vincent Mukwaya, Stephen Mann, Hongjing Dou

**Affiliations:** 1 State Key Laboratory of Metal Matrix Composites, School of Materials Science and Engineering, Shanghai Jiao Tong University, Shanghai, 200240 People’s Republic of China; 2 Max Planck Bristol Centre for Minimal Biology and Centre for Protolife Research, School of Chemistry, University of Bristol, Bristol, BS8 1TS UK

**Keywords:** Biophysical chemistry, Biocatalysis, Biomaterials - cells, Synthetic biology

## Abstract

Although the complexity of synthetic cells has continued to increase in recent years, chemical communication between protocell models and living organisms remains a key challenge in bottom-up synthetic biology and bioengineering. In this Review, we discuss how communication channels and modes of signal processing can be established between living cells and cytomimetic agents such as giant unilamellar lipid vesicles, proteinosomes, polysaccharidosomes, polymer-based giant vesicles and membrane-less coacervate micro-droplets. We describe three potential modes of chemical communication in consortia of synthetic and living cells based on mechanisms of distributed communication and signal processing, physical embodiment and nested communication, and network-based contact-dependent communication. We survey the potential for applying synthetic cell/living cell communication systems in biomedicine, including the in situ production of therapeutics and development of new bioreactors. Finally, we present a short summary of our findings.

## Abstract (web summary)

Synthetic models of cells are becoming increasingly sophisticated, but engineering communication between these and living cells remains challenging. Here the authors review modes of communication and signal processing between living cells and synthetic analogs, such as giant unilamellar vesicles, proteinosomes, and coacervates.

## Introduction

Living cells are soft, wet autonomic micro-devices programmed in the language of chemistry^1^. They operate under non-equilibrium states both at the individual and population levels, are capable of higher-order properties such as compartmentalization, replication, metabolism, and evolution, and exhibit agency via distributed information processing and computation. From a cytomimetic perspective, integrating these complex systems-based properties into synthetic cell models remains a formidable challenge^2–5^. Nevertheless, a growing number of research groups around the world are actively advocating the practical realization of synthetic cell constructs endowed with life-like organization, function, and behavior for modeling the origin of life^3^, assembling cell-free synthetic biological platforms^2,5^ and developing new bio-engineering materials^4^. In this Review, we focus on the ability of protocell models to engage in chemical communication with living cells, highlighting recent breakthroughs and their possible applications. Chemical communication between human cells takes place in three essential modes associated with the endocrine, nervous and immune systems^6^. Firstly, the endocrine system relays molecular communication cues often across extended distances in the form of hormones and growth factors that are deciphered specifically by target cells with corresponding surface or intracellular receptors. As we will discuss, synthetic cells can be engineered to synthesize hormones via cell-free gene expression pathways or produce chemicals that in turn induce the secretion of hormones by neighboring endocrine cells. Secondly, the immune system realizes intercellular communication by contact-based interactions that rely on surface receptors to recognize self and non-self surface markers, or by the release of cytokines to control the growth and activity of other immune system cells. Engineered synthetic cells can intervene in these communication pathways by endogenously synthesizing cytokines or cognate surface receptors and then releasing them locally to target specified immune cells. A third mode of chemical communication between human cells is along nerve tissue, which is electrical, involving membrane depolarization, neurons, synapses, neurotransmitters etc; to date, this pathway has not been interfaced with synthetic cell technology. Cells can also communicate via the direct exchange of RNA; for example, when eukaryotic cells encounter double-stranded RNA (dsRNA), genes carrying a matching sequence are silenced through RNA interference (RNAi)^6^.

We begin the Review with a brief overview concerning the design and construction of soft compartmentalized microsystems that have recently been developed as synthetic cell models capable of chemical communication (Section 2). Such models have been differentiated as *protocell*, *minimal cell*, *artificial cell,* and *synthetic cell* depending on the research context, but these terms are often used interchangeably. Herein, we mainly use the terms *synthetic cell* and *protocell* to describe microscale single-compartment membrane-bounded architectures such as phospholipid-based giant unilamellar vesicles (GUVs), proteinosomes, polysaccharidosomes, and giant polymer-based vesicles, as well as membrane-free molecularly crowded coacervate micro-droplets produced by liquid−liquid phase separation and discrete multi-compartmentalized microstructures with nested microstructures. We discuss the scope for establishing chemical communication in synthetic protocell communities by direct physical contact and integration, or through the diffusive transmission and reception of chemical cues in distributed networks (Section 3). We then analyze how communication channels between synthetic and living cells can in principle be modulated via cognate signaling and information flow involving three modalities: (i) distributed populations and through-space signal processing, (ii) physically assimilated populations (cellular bionics) and nested signaling, and (iii) interfacially connected populations and contact-dependent signaling (Section 4). In each case, we focus on communication modes between soft microscale objects of comparable size. Thus, intracellular signaling between living cells and endocytosed nanoscale constructs such as polymersomes is not addressed (for further details refer to the literature^7–9^). Based on the principles outlined, we survey the potential for applying synthetic cell/living cell communication systems in biomedicine (Section 5). Finally, a short summary is presented (Section 6).

## Synthetic cell modeling

**Fig. 1:**
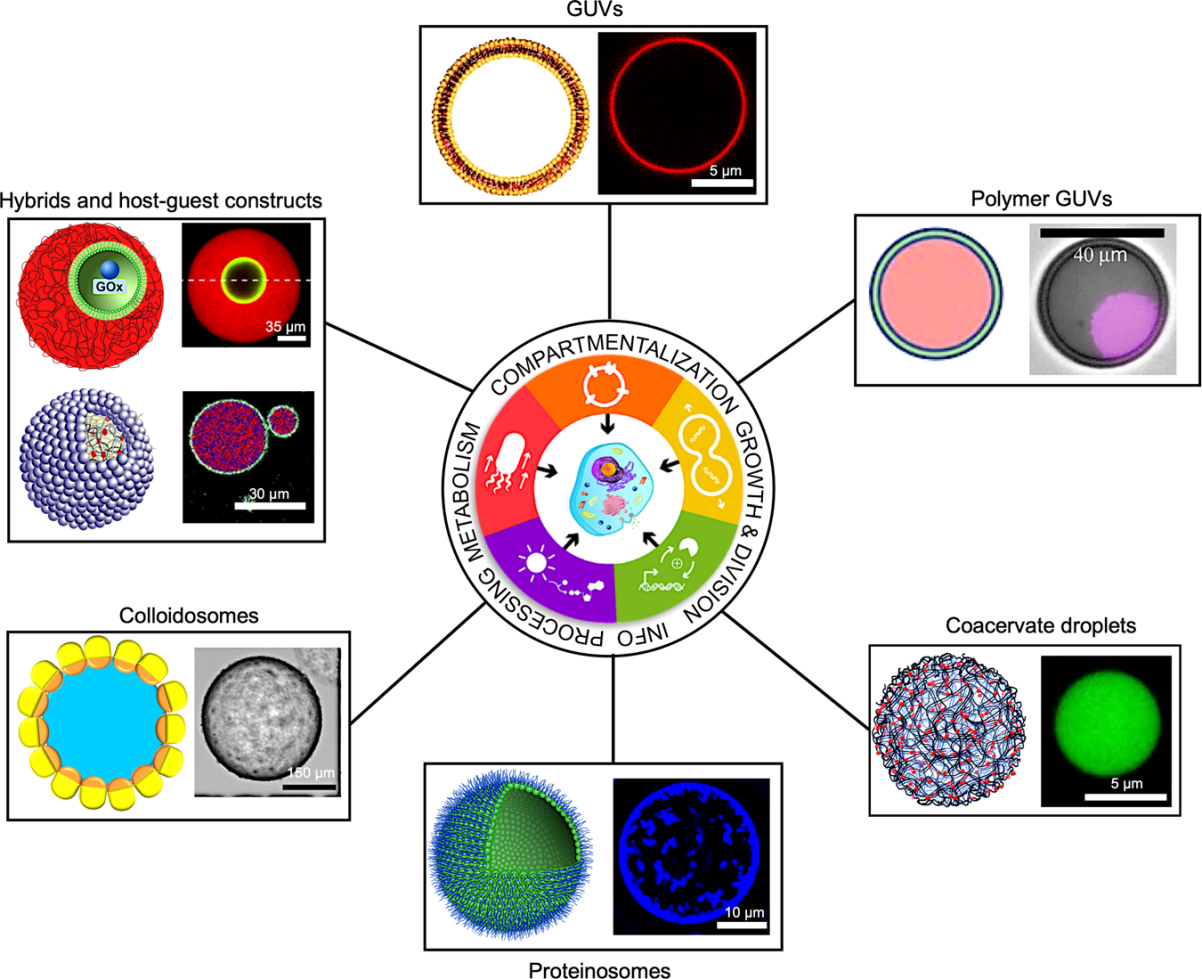
Schematic overview of cell-like synthetic microarchitectures. Diverse soft micro-compartmentalized assemblages are used for synthetic cell modeling. Single and nested compartments are shown as graphical representations (left panels) and experimental microscopy images (right panels). Images were reproduced with permission from^10–17^ Copyright © 2021, American Chemical Society^10^, Copyright © 2016, The Authors^11^, Copyright © 2018, The Royal Society of Chemistry^12^, Copyright © 2013, Nature Publishing Group^13^, Copyright © 2016, John Wiley and Sons^14^, Copyright © 2017, American Chemical Society^15^. Copyright © 2018, The Authors^16,17^. The central cartoon was adapted with permission from Copyright © 2018, The Royal Society Publishing^91^.

Compartmentalization is considered to be indispensable for the origin and operation of living entities, playing fundamental roles in replication, metabolism, and inter- and intracellular communication. It is no surprise therefore that a central tenet in synthetic cell modeling is the concept of semi-permeable compartmentalization, which enables selective diffusion, retention, and release of structural components, chemical energy, enzyme substrates, and informational inputs/outputs (oligonucleotides, promoters/signaling molecules). These attributes can be associated with single-compartment synthetic cells based on GUVs, polymer-based giant vesicles, proteinosomes, polysaccharidosomes, and coacervate micro-droplets or assimilated into multi-level hybrid microstructures with nested architectures (Fig. 1)^10–17^. These cell-like microstructures are usually prepared by the spontaneous self-assembly of molecular or nanoscale amphiphilic building blocks in combination with diverse biological and non-biological components that are captured in the protocell interior or attached to the surface membrane. As a consequence, diverse biomimetic functions such as enzymatic activity, DNA-based information processing, and in vitro genetic programming can be spatially confined and distributed within the soft micro-compartments.

Amongst the increased diversity of cytomimetic models currently available, synthetic cells based on microscale phospholipid vesicles have been the most extensively investigated^18,19^. Biomolecules and biomolecular machinery can be readily encapsulated within the vesicles, giving rise for example to the spatial confinement of reverse transcription and cell-free gene expression^20,21^. As chemical communication with the external environment is often limited by low membrane permeability, porins such as α-hemolysin, OmpF, gramicidin, and DNA nanochannels are incorporated directly into the lipid bilayer during self-assembly or produced endogenously within the vesicles^20,22–24^. Similar strategies have been employed using hydrophobic−hydrophilic synthetic block copolymers to produce water-filled polymer-based vesicles, typically with nanoscale dimensions (polymersomes) and membrane controllable chemistries and permeabilities^17^. The ease of functionalization of the constituent polymer-blocks allows the fabrication of polymersomes with environmentally responsive properties including pH sensitivity^25^, self-bursting^26^, and ligand-specific adhesion^27^. Compared with giant vesicles, protein microcapsules^28^, proteinosomes^13^, and polysaccharidosomes^29^ display increased levels of intrinsic porosity due to the imperfect packing of their constituent amphiphilic protein or polymer-protein/polysaccharide nanoconjugates. Similarly, high levels of intrinsic membrane permeability are observed in inorganic colloidosomes^30^. As a consequence, these constructs are in open communication with most small molecules in their external environment and permeable to short oligonucleotides and macromolecules with molecular masses often less than ca. 40 kDa^31^. Open access can be curtailed to some extent by employing stimuli-responsive building blocks that reversibly contract and swell with changes of temperature or pH^13,29,30^.

Although not strictly a cytomimetic model, membrane-less microdroplets produced by aqueous liquid−liquid phase separation (coacervation/condensation) have emerged as an alternative protocell construct that reproduces the molecularly crowding and diffusional restrictions of living cells^32^. The absence of a membrane facilitates chemical exchange with the surrounding environment while the crowded interior gives rise to selective partitioning of diverse guest entities responsible for biomimetic functions such as cell-free gene expression^33^, photosynthetic activity^12,34^, enzyme-mediated predation^35^ and ribozyme activity^36,37^. A major drawback of these systems stems from the intrinsic instability of the coacervate microdroplets with regard to changes in pH, ionic strength, and temperature, as well as their propensity to undergo coalescence due to low interfacial energies. To circumvent these shortcomings, membrane-coated coacervate droplets have been fabricated to produce integrated cytomimetic models endowed with both a continuous semipermeable shell and molecularly crowded interior^10,15,38–41^. In addition, increasing levels of chemical communication can be achieved by immobilization of the coacervate droplets onto planar surfaces or within hydrogels and exploiting the stationary populations as non-equilibrium spatiotemporal sensors of reaction−diffusion gradients^42–44^.

Assimilation of synthetic cell models into discrete multi-level hybrid microstructures with nested organization provides a step towards combining internal and external modes of chemical communication. From a communication standpoint, the incarceration of micro-compartments within other micro-compartments spatially localizes the receiver and sender microsystems, thereby in principle improving the pathway efficiency, decreasing cross-talk and noise, and providing multiple layers of protection for the signaling network. On the other hand, the presence of multiple barriers can decrease the rates of internalized signal transduction and release of outputs to the external environment due to restrictive and selective molecular diffusion across the different membrane boundaries. These attributes have been exploited to enable incompatible chemical reactions to proceed in parallel within synthetic cells without undesirable crosstalk. For example, partitioning of enzymes in liposomes-in-liposome (vesosomes) constructs containing artificial protein channels has been employed to inhibit deleterious proteolytic reactions, prevent mixing of reactants and products, and facilitate inter-compartment transport and exchange of fluorescent molecules^45^. Similarly, three types of spatiotemporal response to environmental stimuli—inhibited, synchronous or hierarchical—have been chemically programmed into multi-compartmentalized proteinosomes by designing a three-tiered nested structure with spatially and chemically differentiated membranes^46^. In other studies, internal communication pathways involving enzyme-mediated spatial coupling and structural reconfiguration have been implemented in proteinosome-in-coacervate constructs^16^. The nested system functioned co-operatively at low-substrate turnover, while high enzyme activities gave rise to pH-induced disassembly of the droplets, the release of the incarcerated proteinosomes, and self-reconfiguration into spatially organized enzymatically active vesicles-in-proteinosome protocells.

## Communication pathways in synthetic protocell populations

The diversity of available protocell models offers breakthrough opportunities to construct distributed signaling pathways in populations of synthetic cells, opening up the development of interacting cell-like consortia with higher-order functionality. As several comprehensive investigations of communication pathways between synthetic cells have been recently published^17,24,47^, herein we focus on the underlying mechanisms and prerequisites for protocell−protocell communication that in principle provide baseline strategies for the adaption and utilization of synthetic cell/living cell consortia.

Synthetic cells interact with each other with or without feedback via two main communication modes involving direct physical contact and chemical integration, or through diffusive transmission and reception of chemical cues in distributed networks. Communication is dependent on the production and conveyance of molecules originating in sender protocells to receiver protocells that process the signal to generate a quantifiable response or output. The rate of signal transmission is dependent on the spatial separation between the protocells and the nature of their interfaces; for example, whether they are membrane-bound or membrane-free. With the former, changes in semi-permeability influence the encapsulation efficiencies and rates of interchange between vesicles and proteinosomes for example, while membrane-less coacervate droplets provide unfettered access to the environment, high storage potential due to their molecularly crowded interiors, and fast responses to contact-mediated interactions. To increase the selectivity and exchange of information, temperature- or pH-responsive building blocks have been used to construct membrane-gated proteinosomes and inorganic colloidosomes, respectively^13,30,31^, and protein- or DNA-based porins incorporated into liposomes and polymersomes^17,48^.

Signal processing can be implemented within synthetic cell models by inducing endogenous chemical activity via coupled enzyme reactions^49,50^, in vitro gene expression^17,20,24,51^, or DNA strand displacement^23,47,52^. Typically, chemically-based processing of incoming signals involves induction and through-space activation of coupled enzyme reactions between the sender and receiver populations. For example, localized communication within chemically linked populations of glucose oxidase (GOx)- and horseradish peroxidase (HRP)-containing proteinosomes was achieved using a glucose input, hydrogen peroxide signal, and Amplex red-derived readout (resorufin) that produced a spatially distributed chemical output^53^. The localized release of chemical signals can also be used to selectively reconfigure members of a mixed protocell community. For instance, a GOx-mediated hydrogen peroxide signal emanating from a population of silica colloidosomes was used for the in situ membrane re-modeling and re-purposing of a coexistent population of catalytic clay colloidosomes containing alkaline phosphatase^49^. In other studies, the population dynamics of a protocell network were programmed by artificial predator-prey behavior based on antagonistic enzyme-mediated processing^54^. The protocell consortium initially consisted of protease-sensitive GOx-containing killer proteinosomes, non-interacting pH-sensitive, protease-insensitive coacervate droplets containing proteinase K, and proteinosome-adhered pH-resistant coacervate droplets. In the induction stage, glucose-induced production of protons (gluconic acid) within the proteinosomes triggered disassembly of the non-attached protease-containing coacervate droplets. This was followed by the release and transfer of the protease to the pH-resistant proteinosome-attached coacervate droplets, which once weaponized, disassembled the killer proteinosomes via a delayed response-retaliation chemical feedback mechanism.

The use of encapsulated cell-free gene expression systems in vesicle-based synthetic cells has been appropriated for in vitro signal processing and communication. Typically, the strategies are focused on the programmable transfer of membrane-impermeable signaling molecules such as glucose, isopropyl β-*d*-1-thiogalactopyranoside (IPTG), or doxycycline (DOX) via α-hemolysin (α-HL) pores;^20,24,51^ the latter are synthesized endogenously via encapsulated gene circuitry. The approach has been used for example to construct multiple gene cascades within synthetic cells under the control of external signals and inter-vesicular communication^24^, as well as for coupling genetic and enzymatic cascades between vesicles and proteinosomes, respectively^51^. Related approaches have been developed using porous polymer capsules containing a condensed DNA/clay hydrogel core that was accessible to transcription/translation machinery^17^; in this system, synthesized proteins such as RNA polymerases were transferred into neighboring protocells to switch on gene circuits trapped within their hydrogel cores. Given the sensitive concentration dependence of the communication pathway, the methodology served as a rudimentary cytomimetic quorum-sensing system.

Although DNA is not a common currency for intercellular communication in biology, the ability to program the exchange of information by DNA toehold strand displacement reactions offers a promising route to communication and distributed computation in synthetic cell communities. Recently, protocellular consortia that could sense, process, and respond to DNA-based messages was achieved by encapsulating enzyme-free DNA strand displacement circuits inside proteinosomes^47^. As the proteinosomes were semipermeable, input and output signals consisting of single-stranded (*ss*) DNA molecules less than 100 bases in length were free to diffuse between different proteinosome populations. To exchange information within the proteinosomes, the output strand was initially hybridized to a biotinylated double-stranded (*ds*) “gate” oligonucleotide that was anchored inside the protocells by linkage to streptavidin. The passive influx of the input strand from the sender protocells displaced the output strand from the receiver gate complex and resulted in signal processing. As a consequence, cascaded amplification, bidirectional communication, and distributed computational operations were demonstrated within the proteinosome consortia. Moreover, by using photosensitive DNA strands and spatially directed light activation, diffusive signaling gradients could be established between the sender and receiver protocells^52^.

The integration of DNA nanotechnology into synthetic cell models provides a step towards increased levels of programmability and should have a significant impact on developing cognate interactions between populations of protocells and living cells (see Section 4). Besides information processing, DNA nanotechnology has also been employed to construct an artificial signal transduction system to mimic cell−cell communication in binary populations of lipid-based giant membrane vesicles^23^ In this work, a triangular DNA nanoprism (stimulator) with a cholesterol moiety and *ss*DNA side chain was anchored to the lipid membrane of one population of the vesicles, whilst a locked DNA nanopore (actuator) and a triangular DNA nanoprism (receptor) with a *ds*DNA side chain were anchored to the membrane of the second population. Mixing the different vesicles gave rise to a strand displacement reaction between the two types of nanoprisms to release a ssDNA messenger from the receptor. The binding of the released signal to the locked strand of the DNA nanopore, opened up the channel and gave rise to the influx of calcium ions. Mimicking natural signal transduction pathways in this way provides a programmable approach to developing protocell−protocell communication that goes beyond commonly explored mechanisms based on enzyme cascade reactions.

## Chemical communication pathways in synthetic cell/living cell consortia

Living cells decipher communication cues emanating from neighboring cells in their immediate surroundings to trigger collective responses such as quorum sensing, morphogenesis, tissue regeneration, and coordinated motility. In principle, it should be possible to modulate these communication channels via cognate signaling and information flow between synthetic and living cells, thereby bridging the information gap between inanimate and living forms of matter. In general, sustained communication at the living/non-living interface relies on the ability to generate, relay, receive and feedback chemical signals with minimal interference. Considering the range of synthetic cell models and their modes of communication discussed in Sections 2 and 3, differences in membrane composition, structure and activity will strongly influence the strategies adopted for interfacing with living cell populations. For instance, signaling between liposomes and living cells is expected to be attenuated by the low permeability of the vesicle membrane, such that ion channels and membrane porins will be required to attain sufficient levels of material exchange via diffusive transfer through the surrounding milieu. On the other hand, polymer-based membranes with controllable pore sizes that respond to environmental stimuli could provide increased levels of flexibility in relaying signals between populations of living and synthetic cells. Similarly, the utilization of proteins and polysaccharides as building blocks for artificial cell membranes is desirable owing to their intrinsic molecular recognition and catalytic properties which can be harnessed for signal processing in living/synthetic cell networks. Moreover, the reversible assembly/disassembly of coacervate-based protocells can be triggered by external stimuli such as light^55^ to release internally sequestered molecules, suggesting that signal processing networks of biocompatible micro-droplets and living cells might also be possible.

**Fig. 2:**
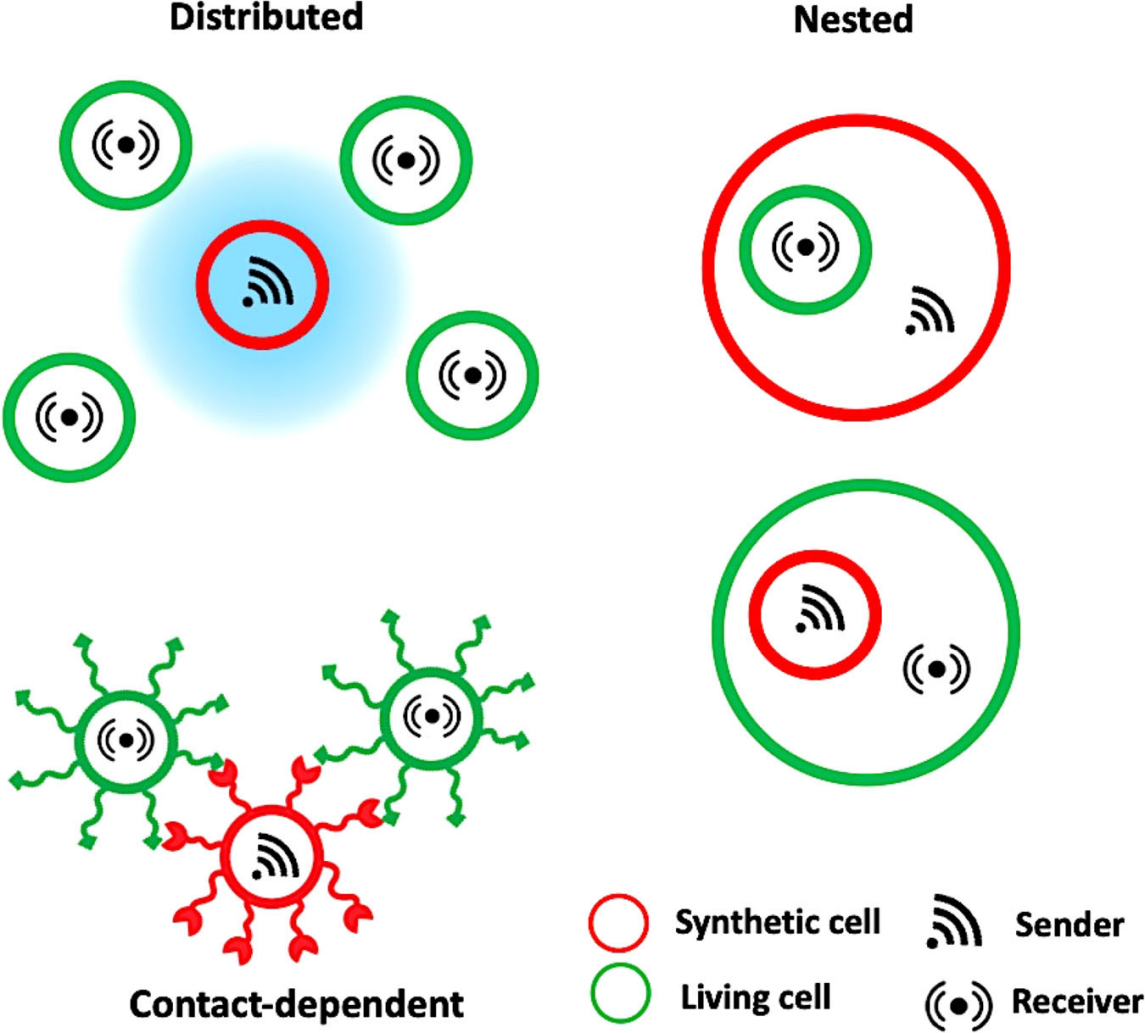
Communication pathways in synthetic cell/living cell consortia. Three modes of chemical communication are shown involving distributed populations and through-space signal processing, nested populations, and embedded signaling, and interfacially connected populations, and contact-dependent signaling pathways. Only unidirectional modes between sender synthetic cells (red) and receiver living cells (green) are shown. Through-space diffusion in the external environment is shaded in blue. Unidirectional pathways between transmitter living cells and receiver synthetic cells as well as bilateral communication with positive or negative feedback are also possible. Adapted with permission from Copyright © 2020, Wiley-VCH GmbH^56^.

In this section, we survey representative examples from the literature that demonstrate the possibility of attaining rudimentary levels of communication in protocell/living cell consortia. As recently described by Elani^56^, communication pathways between synthetic cells and living cells can be implemented by using distributed populations of discrete (non-contact-dependent) entities, physically assimilated populations produced by endosymbiosis, or contact-dependent networks of interfacially coupled populations (Fig. 2).

### Distributed populations and through-space communication

From a practical standpoint, bacterial-based sensing offers the most feasible approach for establishing communication between discrete populations of synthetic and living cells as it provides a wide range of possible signal processing and receiver modules that can be exploited for the cognate exchange of information and materials. Thus, the use of bacteria as a natural partner to protocell models is an attractive proposition as well as offering downstream experimental procedures such as bacterial cultivation, genetic alteration, and phenotypic characterization that are fast and cost-effective. Furthermore, the molecular mechanisms governing gene transcription, translation and regulation are less sophisticated in bacteria than in eukaryotic cells, offering increased scope for both unidirectional and reciprocal communication between the living and synthetic cells. Through-space communication in living/synthetic cell consortia, is likely to be most effective when the following are implemented; (i) signals originate from simple enzymatic, genetic, or chemical processes; (ii) messages are readily transmitted, transduced, and detected; (iii) information is selectively transferable across membranes; (iv) signals are stable in the propagation medium; and (v) detection mechanisms display high specificity to minimize false positives. Living cells also need to be “signal negative” in these pathways so that they can perceive and transduce the signal molecules sent by the synthetic cells in an easily detectable and quantifiable phenotype.

**Fig. 3:**
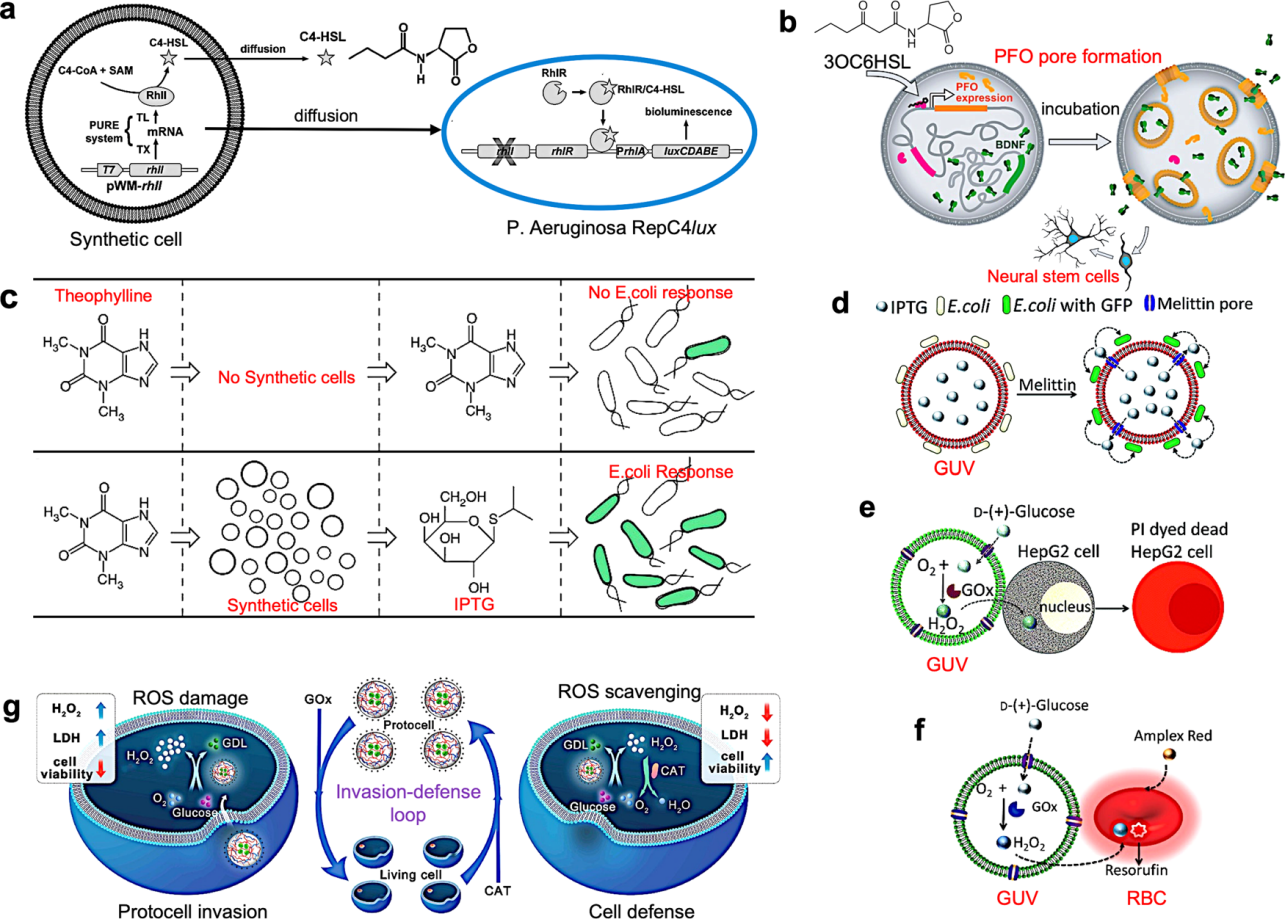
Distributed communication and signal processing. **a** Communication pathway between genetically controlled sender vesicles (synthetic cell) and P. aeruginosa RepC4lux receiver cells. The transmitter system is based on the production of the signal molecule C4-HSL by the synthase RhlI and two precursors (C4-CoA and SAM). The RhlI enzyme is encoded by the rhlI gene (plasmid pWM-rhlI) and produced inside the vesicles by in vitro transcription (TX) and translation (TL) (PURE system). C4-HSL freely diffuses through the vesicle membrane into the medium containing the bacterial cells. The receiver cells contain a genetic reporter device for C4-HSL-induced bioluminescence (PrhlA::luxCDABE) and a mutation inactivating the rhlI gene, so that the living cells cannot produce C4-HSL. C4-HSL binds to receptor RhlR, which in turn triggers luxCDABE transcription by RepC4lux and bioluminescence emission. Adapted with permission from Copyright © 2018, The Royal Society of Chemistry^58^, **b** communication pathway between stimuli-responsive genetically controlled liposomes and neural stem cells. Activation of the AND-gate and expression and assembly of PFO in the vesicle membrane releases BDNF, which in turn induces neural stem cell differentiation. Reproduced with permission from Copyright © 2020, Science^61^. **c** Synthetic cells (circles) translate chemical signals for E. coli (oblongs). In the absence of the vesicles, E.coli cells cannot sense theophylline (top panel). The vesicles are engineered to detect theophylline and in response release IPTG, a chemical signal that induces a response in the E. coli cells (bottom panel). Reproduced with permission from © 2020 WILEY‐VCH Verlag GmbH & Co. KGaA, Weinheim^63^. **d** Schematic showing IPTG-induced GFP expression in E. coli by co-trapped melittin-functionalized IPTG-containing GUVs^22^. **e** Schematic showing H_2_O_2_-induced killing of HepG2 cells by co-trapped melittin-functionalized GOx-containing GUVs. **d** and **e** are reproduced with permission from Copyright © 2019, The Royal Society of Chemistry^22^. **f** Schematic showing chemical signal transduction between a melittin-functionalized GOx-containing GUV (transmitter) and peroxidase-active RBC (receiver). Reproduced with permission from © 2020 WILEY-VCH Verlag GmbH & Co. KGaA, Weinheim^63^. **g** Schematic illustration of the invasion-defense mutual interaction between liquid coacervate microdroplet protocells and living cells. Reproduced with permission from © 2020 WILEY‐VCH Verlag GmbH & Co. KGaA, Weinheim^64^.

These design principles are most readily achieved using distributed populations of sender protocells and receiver bacterial cells. For example, signaling pathways have been established using simple sugars synthesized from a formose reaction encapsulated within vesicles^57^. Passive diffusion of the sugars through the lipid bilayer induced a quorum sensing positive feedback response that gave rise to collective bioluminescence in a receiver population of *Vibrio harveyi* cells. A more generic approach exploits biosensing systems based on acylated homoserine lactones (AHLs), which offer a range of robust chemical communication modules. AHLs are produced by a single enzyme (LuxI-type AHL synthase) in a single-step reaction from an acyl carrier protein and S-adenosyl-methionine. The short-chain AHLs freely diffuse across vesicle and cell membranes, are stable in aqueous media, and trigger a relatively simple signal transduction mechanism involving the activation of an intracellular receptor that directly binds to DNA and regulates transcription. In principle, this genetic signaling pathway can be exploited for establishing both uni- and bi-directional communication modes between living and synthetic cells. For example, the AHL quorum signaling molecule, *N*-butanoyl-L-homoserine lactone (C4-HSL), was enzymatically synthesized inside a giant vesicle-based synthetic cell model and passively released into a population of *Pseudomonas aeruginosa* bacterial cells (Fig. 3a). The binding of C4-HSL to the cell wall receptor RhlR then triggered the expression of the fluorescent protein, mCherry, which was detected by confocal microscopy^58^. To establish bi-directional communication between lipid vesicles and three different types of bacteria, genetic circuits capable of synthesizing several different HSL derivatives in response to chemical signals released from the bacterial cells were incorporated within the protocells^59^. As a consequence, feedback loops were established between the living and synthetic cells or communication pathways implemented between different populations of non-interacting bacteria.

In general, the type and concentration of biomolecules present in the surrounding milieu will affect the efficiency of gene expression within protocells and hence the robustness of signaling pathways between living and synthetic cells. The dependence of synthetic gene circuits on the chemical context results from crosstalk between engineered components, host cells, and environmental dynamics. In this regard, lipid vesicles with encapsulated gene networks have been designed to minimize the sensitivity of the synthetic circuitry to the extracellular chemical conditions^60^. Using an AHL as the primary signaling molecule, the vesicles could detect, interact and activate the self-killing of bacteria in three simulated external environments with different chemical complexity. For example, a unidirectional pathway was established by the protocell-mediated release of the AHL followed by the AHL-mediated gene expression of an antimicrobial peptide within the bacteria.

Communication pathways based on AHLs have also been implemented in mixed populations of protocells and human cells, opening up the possibility of therapeutic applications. For example, stimuli-responsive genetically controlled lipid vesicles were used to chemically communicate with neurons and engineered human embryonic kidney (HEK) 293T cells, as well as promote the differentiation of neural stem cells^61^. The synthetic cell models contained transcription-translation machinery and DNA templates that coded for brain-derived neurotrophic factor (BDNF), LuxR, and perfringolysin O (PFO), a pore-forming toxin. Together, these components constituted a genetic AND-gate that required both LuxR and the inducer molecule N-(3-oxo-hexanoyl)-L-homoserine lactone (3OC6-HSL) for gene expression. On activation of the AND-gate, expression and assembly of PFO in the vesicle membrane resulted in the release of BDNF, which then induced differentiation of murine neural stem cells (Fig. 3b).

Synthetic cell models can be engineered to activate or repress naturally existing sensory pathways in living cells through chemical communication without genetically altering the organism. The small molecule inducer IPTG has been used to expand the sensing range of *Escherichia coli* by translating a silent chemical message into an active signal via intermediate processing using protocells to give a natural cellular response in the *E. coli* cells^62^. This was achieved by encapsulating a genetic program in lipid vesicles that coded for a riboswitch, which activated translation in response to the presence of theophylline. This process then initiated the synthesis of the pore-forming protein α-hemolysin that led to the release of IPTG, which in turn initiated GFP production in the *E. coli* population. As a consequence, the *E. coli* cells, which naturally do not respond to theophylline became active when theophylline was added because of signal processing by the vesicles (Fig. 3c). A similar communication pathway between an acoustically co-trapped binary population of IPTG-containing giant unilamellar vesicles (GUVs) and *E. coli* cells has also been demonstrated^22^. In this case, the IPTG signal was released from the GUVs upon the addition of the pore-forming protein, melittin (Fig. 3d).

In contrast to gene-based pathways, communication modes between dispersed populations of living and synthetic cells that encompass signals derived from purely chemical (enzyme) activities are relatively easier to construct but lack high levels of programmability. However, they may be useful when definitive responses are required in the receiver living cells. For example, GUVs containing a glucose oxidase/horseradish (GOx/HRP) enzyme cascade reaction were effective in killing HepG2 cancer cells by transfer of hydrogen peroxide (H_2_O_2_) from the synthetic to living cells in the presence of a glucose input (Fig. 3e)^22^. Replacing the HepG2 cells by red blood cells utilized the GUV-derived H_2_O_2_ signal for the induction of peroxidase activity in the co-trapped erythrocytes (Fig. 3f)^63^. Similarly, antagonistic enzyme reactions have been employed to develop an invasion-defense loop between HepG2 cells containing catalase (H_2_O_2_ scavenging) and GOx-containing (H_2_O_2_ producing) coacervate-based protocells in the presence of glucose (Fig. 3g)^64^.

Taken together, the above studies highlight the potential for using synthetic cell constructs as platforms for the in-situ synthesis and on-demand release of biochemical signals that elicit desired phenotypic changes in bacterial and to a lesser extent in eukaryotic cells. It seems reasonable to propose that future studies on synthetic/living cell communication pathways in dispersed populations will continue to develop more elaborate feedback loops and sophisticated circuitry; for example, by using negative feedback processes to regulate bacterial populations via protocell-mediated quorum quenching.

### Nested populations and cellular bionics

The construction of embodied architectures in which living and synthetic cells are functionally and structurally intermingled offers exciting opportunities for the fabrication of cellular bionic systems^65–67^. The physical integration of living cells and protocell models produces host-guest constructs with new functional (non-native) modules, hierarchical organization, and increased complexity, which together facilitate the implementation of nested communication pathways. For example, endocytosis of synthetic cells within living cellular hosts provides a step towards the integration of artificial communication channels into natural metabolic and genetic networks. Alternatively, entrapment of living cells in protocellular hosts such as GUVs, proteinosomes, or coacervate micro-droplets enables the construction and spatial organization of microscale cellular colonies housed within specialized micro-environments.

**Fig. 4:**
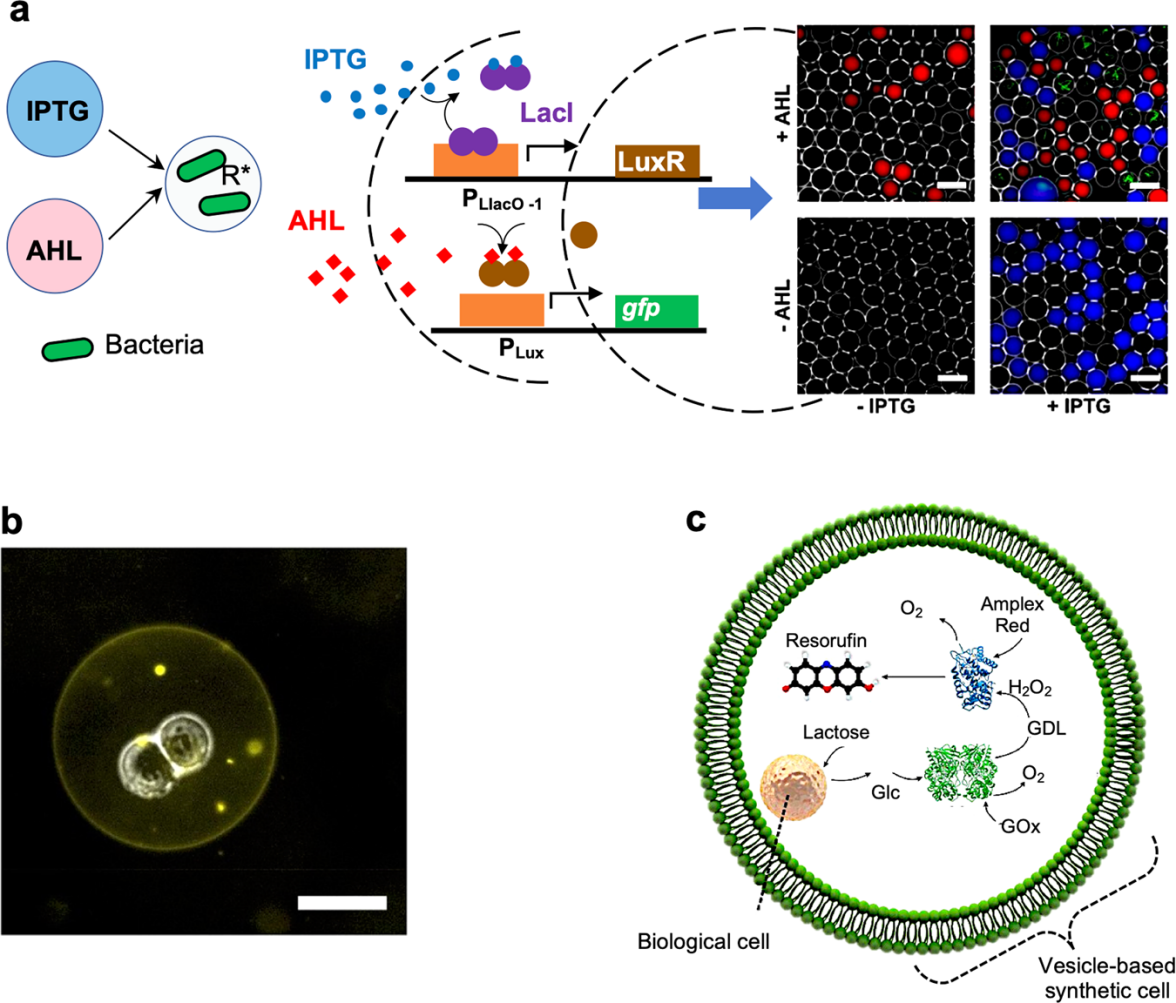
Nested communication and cellular bionics. **a** Inducers 3OC6-HSL (AHL) and IPTG diffuse from reservoir droplets to receiver droplets (R^*^) containing engineered bacteria (left panel). The bacteria contain a genetic AND gate (center panel) with a fluorescence output. Fluorescence microscopy images arranged in a truth table (right panel); scale bars = 50 μm. Reproduced with permission from, Copyright © 2014, American Chemical Society^65^. **b** Brightfield/fluorescence microscopy composite image showing a cellular bionic system comprising a single host lipid vesicle and two incarcerated BE colon carcinoma cells. Scale bar = 25 µm. Reproduced with permission from, Copyright © 2018, Springer Nature^67^. **c** Schematic of a single vesicle-based synthetic cell containing colon carcinoma cells and a GOx/HRP enzyme cascade. Cell-mediated production of glucose (Glc) in the vesicle lumen switches on GOx activity to release hydrogen peroxide and D-gluconolactone (GDL) followed by HRP-mediated peroxidation of Amplex Ultra Red to give a fluorescent output (resorufin) inside the synthetic cells. Reproduced with permission from, Copyright © 2018, Springer Nature^67^.

An effective test system of nested communication is the use of picolitre-sized water-in-oil emulsion droplets to compartmentalize bacteria and spatially organize the consortia. For example, the response of emulsion droplet-encapsulated bacteria to small diffusible inducer molecules such as IPTG and 3OC6-HSL has been investigated^65^. Diffusion of the inducers through the oil medium enabled sender bacteria in one droplet to communicate with receiver bacteria in neighboring compartments. Significantly, gene expression of GFP and the resulting fluorescence output was switched on at high levels if the bacteria were equipped with a genetic AND gate that operated in the presence of both the IPTG and 3OC6-HSL (Fig. 4a). Moreover, linear arrangements of similar microdroplet compartments were used to investigate spatially distributed gene expression in the droplet-entrapped bacteria^66^. The quasi-one-dimensional geometry allowed for better control of the boundary conditions, facilitated a more straightforward analysis of the experiments, and demonstrated a higher level of mutual coupling of the neighboring compartments. These studies pave the way towards the design of directional communication pathways between synthetic and living entities that exhibit minimal dampening of the chemical signals when propagated over relatively long distances.

Entrapment of living cells in protocellular hosts such as vesicles or coacervate micro-droplets provides a step towards the construction of hybrid modules comprising discrete cellular micro-environments that can be interfaced with the external milieu. For example, living cells have been encapsulated into large lipid vesicles to produce a chemically and physically linked cellular bionic systems with symbiotic properties (Fig. 4b)^67^. The vesicle membrane acted as a protective barrier to toxic cations in the external environment while incarcerated colon carcinoma cells served as bioreactors to process chemicals specifically within the vesicle lumen^67^. The cells were genetically modified to express β-galactosidase, which converted lactose into glucose, releasing the glucose into the GUV lumen and switching on an enzyme cascade (Fig. 4c) such that cellular and non-cellular chemical processing were coupled within the nested module. Using a similar strategy, genetically engineered *E. coli* cells capable of detecting carcinoma-produced lactate were encapsulated within GUVs to produce a nested cytomimetic system exhibiting enhanced biosensing^68^.

As the above strategies depend on the successful entrapment of living cells during assembly of membrane-bounded vesicles, they can be compromised by the use of organic solvents, low encapsulation efficiencies, and limited numbers of viable cells. In contrast, the use of biocompatible membrane-less coacervate micro-droplets produced by aqueous two-phase separation provides in principle a more facile route to the capture and assimilation of living cells^69,70^. Although such systems have not been used so far as communication platforms, and are prone to coalescence and concomitant loss of the nested micro-architecture, they can be stabilized, suggesting potential uses in future studies. For example, densely packed micro-spheroids containing photosynthetically active algal or algal/bacterial cell populations have been assembled using dextran-in-PEG aqueous emulsion droplets and exploited as living/synthetic cellular devices for the generation of oxygen or hydrogen under daylight^34^.

### Contact-dependent communication

Contact-dependent modes of communication involve interfacially connected synthetic and biological cells organized within particular spatial arrangements. In principle, communication occurs through diffusible local chemical signals acting on cognate membrane receptors that produce signaling cascades. To date, there are only a few reports on interfacially coupled synthetic/living cell consortia that serve as communication platforms. In contrast, physically and chemically attached arrangements of mixed populations of synthetic cells have been developed as prototissues exhibiting collective modes of light-activated gene-expression^71^, controlled prodrug activation and release^72^, and temperature-mediated chemo-mechanical transduction^53^.

**Fig. 5:**
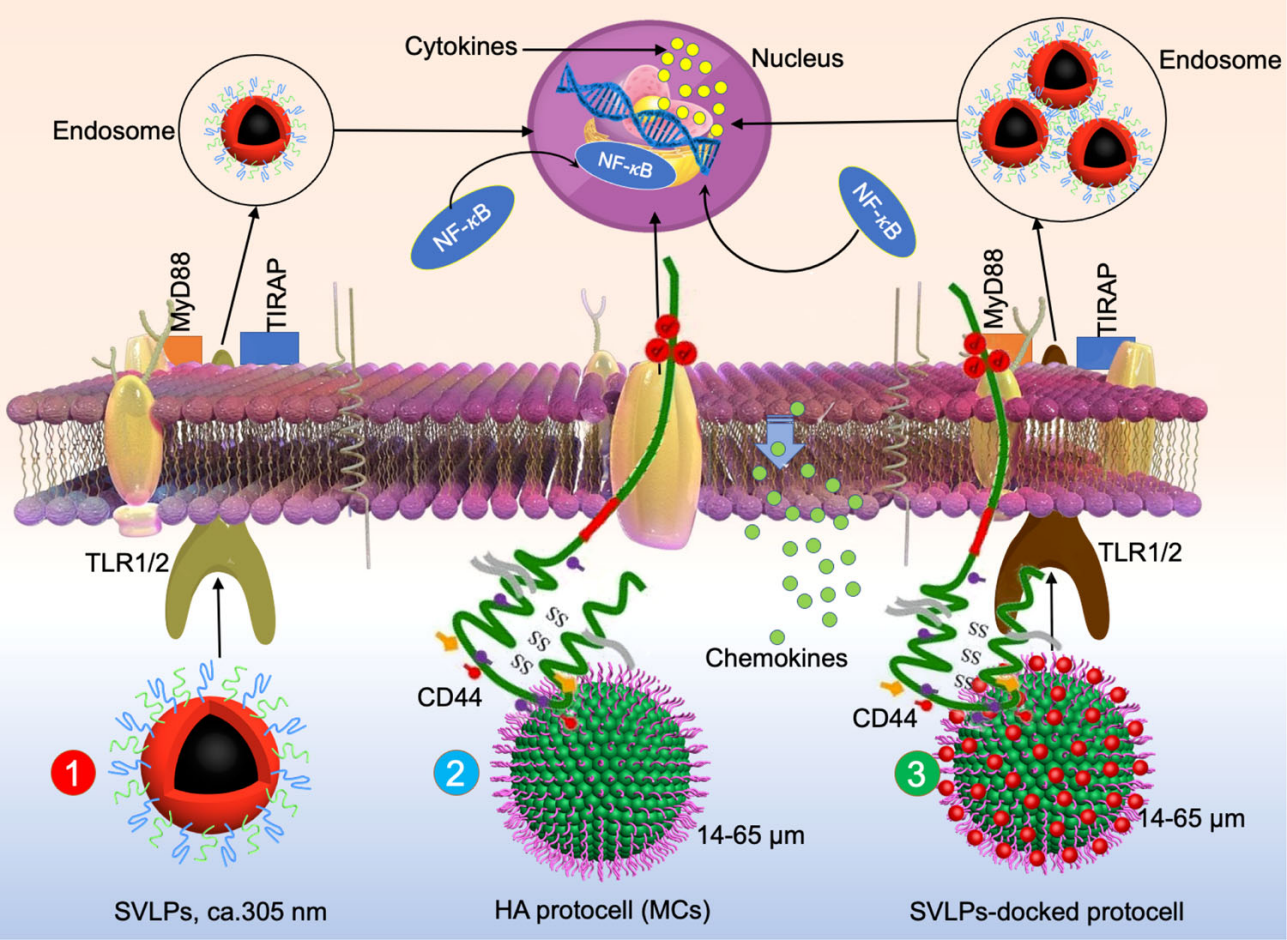
Contact-dependent communication between the living and synthetic cells. Summary scheme depicting ligand/receptor interactions responsible for synergistic macrophage activation in the presence of SVLP-presenting polysaccharidosomes (**3**). Pathways for unattached SVLPs (**1**) and non-functionalized polysaccharidosomes (**2**) are also depicted. Reproduced with permission from Elsevier^76^.

New approaches to contact-dependent communication between the living and synthetic cells could be forthcoming by alignment with current research on artificial antigen-presenting constructs. Although the investigation of changes in the size, shape, and surface-ligand functionalization of nanosized artificial antigen-presenting materials has been extensively investigated—for example as immunomodulation devices—studies on microscale antigen-presenting entities have focused primarily on the use of polymer^73,74^ and inorganic microparticles^75^ rather than synthetic cell models such as GUVs and proteinosomes. In general, the particle-based systems act by inducing T-cell proliferation and secretion of chemokines via signaling systems involving a peptide-loaded major histocompatibility complex (p-MHC) and co-stimulatory molecule such as anti-CD28. It seems feasible that these key attributes could be integrated into micro-compartmentalized systems as a step towards establishing contact-dependent signaling between synthetic cell models and living cells. In this regard, immunogenic polysaccharidosomes have been constructed using hyaluronic acid (HA)/polymer nanoconjugate membrane building blocks^76^. Low molecular weight HA was chosen as the HA chains activate the innate immune system via interactions principally with CD44 membrane receptors. As a consequence, the polysaccharidosomes achieved moderate levels of activation towards MH-S macrophages in vitro. Moreover, the immunogenic response was further enhanced by embellishing the protocells with surface-attached synthetic virus-like particles (SVLPs) that were co-functionalized with a lipopeptide Toll-like receptor (TLR2) agonist (Pam3SK4) and a cell membrane binding lectin (wheat germ agglutinin, WGA)^76^. The macrophage hyper-activation was attributed to a synergistic three-component multi-receptor process involving HA and CD44, Pam3SK4 and TLR2, and WGA and sialic acid-rich membrane glycoproteins (Fig. 5).

In summary, although current protocell technologies cannot match the level of sophistication attained in biological signaling pathways, significant advances have been made in limited numbers of synthetic/living cell consortia via the three communication modes described above. At this juncture, most communication pathways between synthetic cells and living entities have been with single-celled organisms, particularly bacteria, with only a few reports on interactions with mammalian cells^61^. We expect further advances in this direction to be forthcoming, especially when focused on the integration of antigen-presenting technologies into synthetic cell models. This in turn, will offer increased scope to regulate the collective behaviors of human cells by cognate synthetic cell partners, and provide wide-ranging opportunities for developing applications in numerous areas as discussed in the next section.

## Therapeutic applications at the synthetic/living cell interface

Physical entrapment of mammalian cells within enclosed capsules or hydrogel microspheres is now commonplace in therapy, tissue engineering, and biomanufacturing applications. Conversely, the use of synthetic cell models as programmable micro-compartmentalized agents for interfacing with living cells is at a comparably early stage of development^77^. Based on the above discussions, we foresee an expanding niche for the utilization of protocells as cellular communication devices, implants, and cytomimetic partners optimized for the protection and regulation of living cells. This approach remains at an early stage of development, and criteria such as the signaling flow, circumvention of synthetic and biological barriers, preservation of in situ activity, biocompatibility, targeting, degradation profiles, and scalability will need to be addressed.

**Fig. 6:**
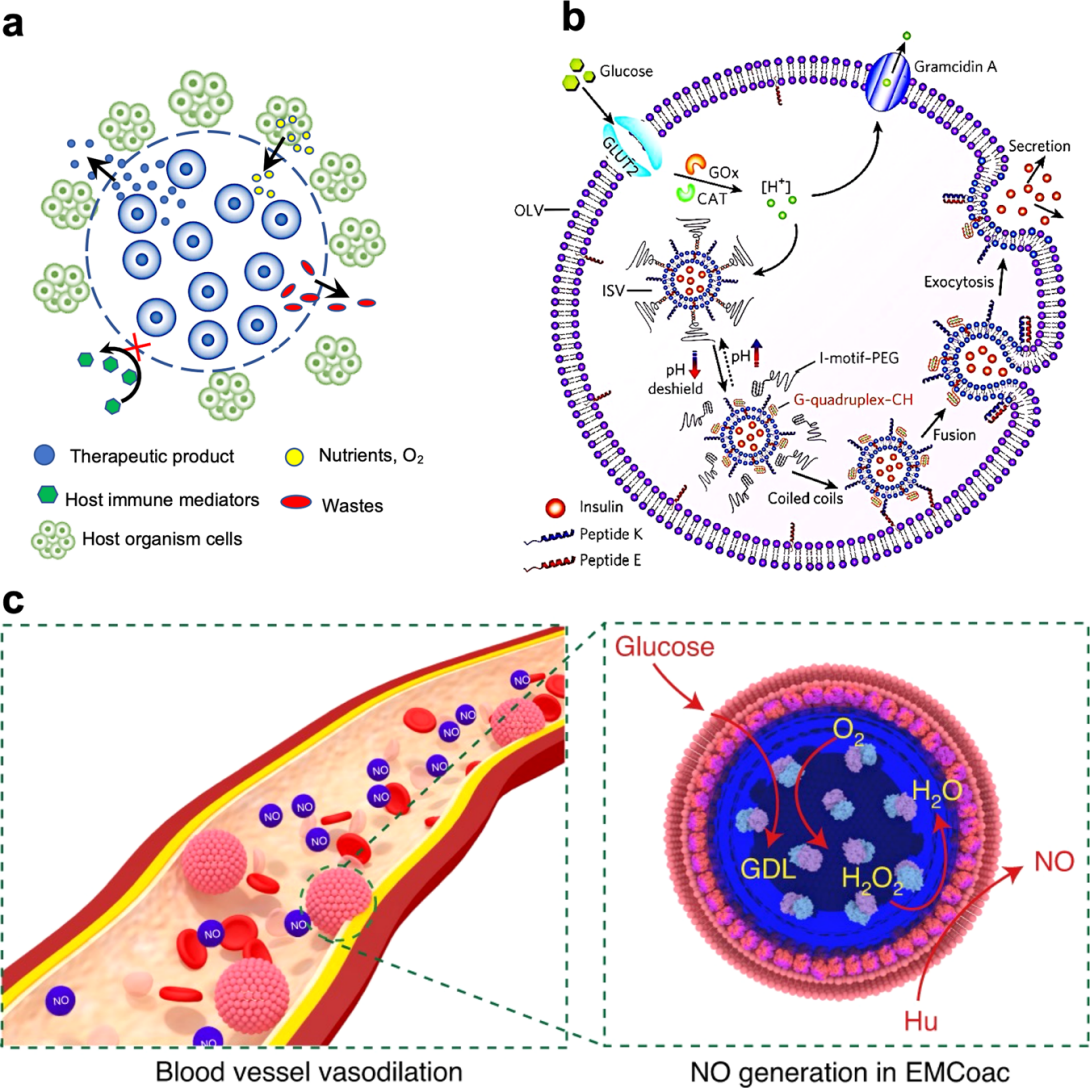
Therapeutic applications at the synthetic/living cell interface. **a** Schematic showing cell encapsulation within synthetic membranes or hydrogel microcapsules (dashed circle) to produce cellular bionic systems with therapeutic potential. Therapeutic cells are shown in blue. Adapted with permission from Copyright © 2018 John Wiley and Sons^79^. **b** Schematic of a therapeutic synthetic cell (AβC) with a glucose metabolism system and membrane fusion machinery that is coupled to the external release of insulin by programmed exocytosis of internalized insulin-loaded vesicles (ISVs). GOx, glucose oxidase; CAT, catalase; square brackets denote concentration. Reproduced with permission from Springer Nature^87^. **c** Illustration showing in vitro and in vivo GOx/Hb cascade generation of NO at micromolar concentrations in the presence of coacervate-sequestered enzyme substrates (glucose and hydroxyurea, respectively) as a step towards protocell-mediated blood vessel vasodilation. Hb and GOx are spatially positioned on the periphery and in the interior of the protocell bioreactor, respectively. GDL, D-gluconolactone; Hu, hydroxyurea. Reproduced with permission from Springer Nature^90^.

Currently, only a limited number of studies showcase how chemical communication pathways between living and synthetic cells can be harnessed for medical applications. In principle, the synthesis of biologically relevant molecules inside protocells and their conveyance across semi-permeable membranes to induce programmable changes in cognate living cells offers opportunities in immunology, regenerative medicine, and replacement therapy. Embedding living cells in synthetic micro- or macro-sized enclosures to produce nested cellular bionic systems is beneficial in cell therapy as it allows the use of allogenic cells for transplantation^78^. The encapsulated cells are protected from the host immune system, thereby reducing the need for the administration of anti-immunoresponse drugs and providing a sustained release of therapeutic agents (Fig. 6a)^79^. Typically, cell-containing alginate, collagen, or Matrgel capsules are used to restore and regulate insulin supply (type I diabetes)^80,81^, provide spatial and chemical immuno-isolation of xenogeneic hepatocytes, and bone marrow stromal cells (liver tissue engineering)^82–84^, and promote osteogenesis and bone repair^85,86^. Based on this burgeoning particle-based biotechnology, it should be possible to develop similar strategies employing synthetic cell models with their increased range of cytomimetic functions. In this regard, a pioneering example of a therapeutic synthetic cell system was reported by Chen and co-workers who constructed phospholipid-based artificial beta cells (AbCs) with a multicompartmentalized vesicles-in-vesicle nested structure containing a glucose metabolism system and membrane fusion machinery (Fig. 6b)^87^. The AbCs could distinguish between high and normal glucose levels through a sequential cascade involving glucose uptake, enzymatic oxidation, and proton efflux. Under hyperglycemic conditions, high glucose uptake and oxidation generated a low pH inside the synthetic cells, which in turn induced the steric deshielding of peptides tethered to the external surface of insulin-loaded small liposomal vesicles present within the protocell. As a consequence, the membrane-bound peptides underwent coiled-coil interactions with complementary peptides anchored to the inner surfaces of the synthetic cell, thereby promoting fusion and exocytosis, leading to insulin release from the AbCs.

Vesicles containing transcription/translation systems have been exploited for potential applications in cancer therapies. For example, proteoliposomes capable of internally expressing a membrane-inserted voltage-dependent anion channel (VDAC) and pro-apoptotic proteins (Bak) were endocytosed in mammalian cells and used to induce apoptosis^88^. Endogeneous expression of VDAC and Bak resulted in in situ integration of the proteins into the lipid bilayer and activation of the apoptosis pathway as demonstrated by the increase in intracellular levels of cytochrome *c* and caspases. In other studies, therapeutic proteins were synthesized inside tumors by using lipid vesicles containing the requisite molecular machinery for the gene expression of anticancer proteins^89^. Significantly, the synthetic cells sourced nutrients from the biological microenvironment to trigger protein synthesis within the vesicles. As a consequence, culturing 4T1 breast cancer cells in the presence of vesicles encoded to secrete *Pseudomonas* exotoxin A (PE) resulted in the killing of most of the malignant cells. Moreover, histological evaluation of the tumor tissue of 4T1 tumor-bearing mice revealed that extensive carcinoma apoptosis ensued after local injection of the PE-producing protocells.

Interfacing synthetic cell constructs with signaling pathways in cellular tissues has recently been demonstrated using hybrid protocells with high hemocompatibility and increased blood circulation times^90^. The synthetic cells were produced by self-assembly of hemoglobin (Hb)-containing red blood cell membrane fragments on the surface of polysaccharide/polynucleotide coacervate micro-droplets containing glucose oxidase (GOx). The sequestered enzymes generated a protocell-mediated flux of nitric oxide (NO) in the presence of glucose and hydroxyurea, which in turn activated a complex process of signal transduction that resulted in blood vessel vasodilation in vitro and in vivo, providing a first step towards therapeutic development (Fig. 6c).

## Outlook

Advances in synthetic biology, materials science, and computing over the past 50 years have accelerated progress in the field of artificial life through the design and construction of cell-like microarchitectures via both bottom-up and top-down methods. Cytomimetic self-assembled structures have been constructed in the form of GUVs, giant polymer vesicles, proteinosomes, polysaccharidosomes, colloidosomes, and coacervates micro-droplets, as well as hybrid combinations of these micro-compartmentalized systems. Typically, biomimetic functions such as enzyme cascades, growth and division, motility, predation, phagocytosis, cell-free gene expression, membrane-gating, and chemical communication have been demonstrated. However, the sophistication of these synthetic protocellular systems is rudimentary compared with the functional integration of living cells, and there remain many challenges before life-like properties are emulated.

As alluded to in this review, the bottom-up approach to synthetic cell construction is a promising step towards the microscale optimization and integration of biological and biomimetic processes in vitro and to a lesser extent in vivo. This approach has undoubtedly improved the complexity of synthetic cell design in terms of bio-catalytic potential, chemical signaling, and information processing but remains limited in terms of efficiency and self-sufficiency. More generally, the realization of higher-level processes in synthetic cell models such as autonomy, self-replication, and autopoiesis (self-production) remain elusive. However, as described in this review, considerable progress has been made albeit at a rudimentary level with regard to chemical communication in protocell/protocell and synthetic cell/living cell communities. Currently, the advances are focused primarily on the interface with bacterial cells because the signaling and transduction pathways are considerably more complex in eukaryotic cells. In this regard, the first steps towards the fabrication of synthetic cells with mammalian cell cognition require the incorporation of receptors in synthetic cell membranes, utilization of building blocks with intrinsic living cell-cognate properties, and the encapsulation of chemical and enzymatic reactions necessary for the synthesis of relevant signaling agents or modulation of communication pathways. We hope that this review will help to broaden the repertoire of synthetic/living cell systems to embrace cognate interactions with mammalian cells for advancing future opportunities in cellular bionics, therapeutics, immunology, and regenerative medicine.
